# Mobile Location with NLOS Identification and Mitigation Based on Modified Kalman Filtering

**DOI:** 10.3390/s110201641

**Published:** 2011-01-27

**Authors:** Wei Ke, Lenan Wu

**Affiliations:** 1 School of Information Science and Engineering, Southeast University, Nanjing 210096, China; E-Mail: wuln@seu.edu.cn; 2 School of Physics and Technology, Nanjing Normal University, Nanjing 210097, China

**Keywords:** extended Kalman filter (EKF), mobile location, LOS, NLOS, identification

## Abstract

In order to enhance accuracy and reliability of wireless location in the mixed line-of-sight (LOS) and non-line-of-sight (NLOS) environments, a robust mobile location algorithm is presented to track the position of a mobile node (MN). An extended Kalman filter (EKF) modified in the updating phase is utilized to reduce the NLOS error in rough wireless environments, in which the NLOS bias contained in each measurement range is estimated directly by the constrained optimization method. To identify the change of channel situation between NLOS and LOS, a low complexity identification method based on innovation vectors is proposed. Numerical results illustrate that the location errors of the proposed algorithm are all significantly smaller than those of the iterated NLOS EKF algorithm and the conventional EKF algorithm in different LOS/NLOS conditions. Moreover, this location method does not require any statistical distribution knowledge of the NLOS error. In addition, complexity experiments suggest that this algorithm supports real-time applications.

## 1. Introduction

In order to understand sensor data in spatial context or for proper navigation throughout a sensing region, automatic location of the sensors in wireless networks is a key enabling technology. Therefore, mobile location technologies, which are designated to estimate the position of a MN, have drawn a lot of attention for its various potential location-based services [1,2]. Although Global Positioning System (GPS) has been in service for many years, it is only available in GPS-enabled devices and may encounter problems in urban and indoor environments. The poor signal penetration capabilities of the GPS prevents consumer-grade GPS receivers from making reliable measurements in certain urban settings and indoor environments where there are no direct visibilities to satellites [3]. Thus, the location estimation based on existing wireless infrastructures has advanced rapidly in recent years. Research interests have been further aroused after the US Federal Commission Committee (FCC) has requested that the location accuracy of the emergency calls should be 100 m for 67% of time and 300 m for 95% of time for the network-based location systems.

If the LOS propagation exists between the MN and all fixed nodes (FNs), which are known-location reference anchor nodes in a sensor network, a high location accuracy can usually be achieved using the conventional location algorithms [4]. However, since the direct path from the MN to a FN can be blocked by buildings and other obstacles, the transmitted signal could only reach the receiver through reflected, diffracted, or scattered paths called NLOS paths. The NLOS propagation generally leads to a positive bias in the estimation range and causes a serious error in the mobile location estimation. The problem of containing positive bias is even recognized as a “killer” issue in the location estimation [5,6], which is also the main focus of this paper.

In the open literature, many methods have been employed to mitigate the adverse effect. Generally, these methods can be divided into two categories: methods for static positioning systems and methods for mobile tracking systems. Reference [7,8] have summarized the methods for static position systems. However, these methods are not effective for mobility tracking systems, in which redundant measurements in time series can be exploited. Recently, the Kalman filtering techniques are applied for range measurements smoothing and NLOS error mitigation. Although some novel filtering techniques such as unscented Kalman filtering and particle filter are superior to the EKF for nonlinear systems [9], the EKF is one of the most widely used methods for tracking and estimation due to its simplicity, optimality, tractability and robustness. The EKF-based algorithms are suggested in [10,11] as a promising alternative to range measurement for smoothing and mitigating NLOS error. The identification of LOS/NLOS conditions in [10,11] is based on the reasonable assumption that the standard deviation of the range measurement in the case of NLOS is significantly larger than that of the LOS case. But the deviation threshold is set manually according to the experiments, which is difficult to be chosen in the diversified environments. A Kalman based IMM smoother [12,13] is proposed to estimate the range between the MN and the corresponding FN in the mixed LOS/NLOS conditions. This method can track the true range distance more accurately than the rough LOS/NLOS smoother in [10,11], especially in the transitional intervals. But in this method, the IMM KF range smoothing and the geolocation positioning are two separated steps with each introducing estimation error, which lead to a larger resultant location error. The method in [14] proposed a one-order hidden Markov chain to simultaneously model the transition of the LOS/NLOS condition and the MN position. However, the grid based Bayesian estimation is complex for computing.

Although the above algorithms mitigate the influence from the NLOS errors and improve the location accuracy in a certain extent, the more thorough method is directly to estimate and eliminate the NLOS bias in each measurement. Reference [15] has presented a modified Kalman tracker which increases the dimension of the state vector by adding the NLOS bias as additional parameters to be estimated. The NLOS bias estimation provided for this new Kalman tracker improves the performance of the tracking position in NLOS scenarios. Although this method is not always effective for mobile tracking systems due to lack the proven research in theory, it inspired us to study the new EKF tracking algorithm combining with direct NLOS bias estimation. Different from the previous works, our method firstly estimates the NLOS errors using nonlinear optimization under the constraint of positive biases and geometrical relationship. Since all those NLOS distances can be converted into approximate LOS distances by eliminating the NLOS bias in the updating process, the EKF can be applied to achieve a high estimation precision. To reduce the unnecessary computing time when the LOS situation appears, a low complexity method based on innovation vectors is utilized to identify the LOS/NLOS conditions within the EKF framework. This algorithm does not require any priori statistical information of the NLOS error, and computation complexity is still low.

The remainder of this paper is organized as follows. The measurement model is described in Section 2. The NLOS bias estimation using constrained optimization method is developed in Section 3, and Section 4 formulates the modified EKF-based tracking algorithm with NLOS correction. Section 5 presents the LOS/NLOS identification method for decreasing computing time. The simulation results and performance analysis are discussed in Section 6. Finally, conclusions are drawn in Section 7.

## 2. Measurement Model

Assume that sensors are capable of transmission and reception, and then unknown-location devices are able to make measurements to multiple reference nodes. In the existing wireless systems, the range between the MN and a known-location FN could be measured by time of arrival (TOA), round trip time, and signal strength measurement techniques. In this study, we assume the mobility estimation is based on TOA at the MN, and the distance measurement is obtained by multiplying the time measurement by the speed of light. Assume that the MN has detected the range signals from the *M* FNs, and then the range between the MN and the *i*th FN at the time k can be modeled as:

(1) $$z_{i}(k)=d_{i}(k)+n_{i}(k)+b_{i}(k)\;,\;i=1,2\cdots M$$

where:

(2) $$d_{i}(k)=f_{i}[\text{X}(k)]=\sqrt{{\left(x_{k}-x_{i}\right)}^{2}+{\left(y_{k}-y_{i}\right)}^{2}}\;,\;i=1,2\cdots M$$

is the true range between the *i*th FN and the MN, **X**(*k*) = [*x*_*k*_, *y*_*k*_]*^T^* represents the unknown coordinates, and [*x*_*i*_, *y*_*i*_]*^T^* is the *i*th FN location. *n*_*i*_(*k*) and *b*_*i*_(*k*) represents the measurement noise and the effective NLOS component, respectively. The term *n*_*i*_(*k*) is modeled as an independent and identically distributed zero-mean white Gaussian process with covariance matrix **R**(*k*). The term *b*_*i*_(*k*) ≥ 0 always makes a TOA measurement longer than the true distance, whose probability distribution is generally unknown beforehand. Equation (1) can be written in a compact form as follows:

(3) z(k)=d(k)+n(k)+b(k)

where:

(4) $$\text{z}(k)={\left[z_{1}(k),z_{2}(k),\cdots z_{M}(k)\right]}^{T}\;$$

(5) $$\text{d}(k)={\left[d_{1}(k),d_{2}(k),\cdots d_{M}(k)\right]}^{T}$$

(6) $$\text{n}(k)={\left[n_{1}(k),n_{2}(k),\cdots n_{M}(k)\right]}^{T}$$

(7) $$\text{b}(k)={\left[b_{1}(k),b_{2}(k),\cdots b_{M}(k)\right]}^{T}$$

(8) $$\text{f}(\text{X})={\left[f_{1}(\text{X}),f_{2}(\text{X}),\cdots f_{M}(\text{X})\right]}^{T}$$

## 3. Direct NLOS Bias Estimation

Here, we propose a least squares (LS) optimization-based technique to estimate the NLOS propagation delay without requiring any prior statistics information. With the linearization of the system using Taylor’s series approximation as discussed in [16], a linearized measurement vector derived from (3) is defined as follows:

(9) $$\text{y}(k)={\text{H}}_{0}\text{X}(k)+\text{n}(k)+\text{b}(k)$$

where **H**_0_ is the Jacobian matrix of **f**(**X**) at a reference point **X**_0_, *viz*.

(10) $${\text{H}}_{0}={\left[\begin{array}{cccc} \frac{\partial d_{1}(k)}{\partial x} & \frac{\partial d_{2}(k)}{\partial x} & \cdots & \frac{\partial d_{M}(k)}{\partial x}\\ \frac{\partial d_{1}(k)}{\partial y} & \frac{\partial d_{2}(k)}{\partial y} & \cdots & \frac{\partial d_{M}(k)}{\partial y} \end{array}\right]}_{\text{X}={\text{X}}_{0}}^{T}$$

Note that the reference point **X**_0_ should be chosen close enough to the true position in order for (9) to be valid. The reference point coordinate estimates may be determined using the simple estimator as follows. Because the MN cannot be located farther than *z*_*i*_(*k*) from the *i*th FN, therefore, it must be located within a circle of radius *z*_*i*_(*k*) centered around the *i*th FN. By repeating these arguments for all FNs, and then their circles are determined; so their intersections define the set of the possible location points for the MN. As a result, the reference estimate **X**_0_ = (*x*^(0)^, *y*^(0)^) for the MN location can be obtained by finding the center of the feasible region. With three FNs, for example, it is calculated by averaging the coordinates of the points of intersection (*x*^(1)^, *y*^(1)^), (*x*^(2)^, *y*^(2)^) and (*x*^(3)^, *y*^(3)^), *i.e.* (*x*^(0)^ = (*x*^(1)^ + *x*^(2)^ + *x*^(3)^)/3 and *y*^(0)^ = (*y*^(1)^ + *y*^(2)^ + *y*^(3)^)/3. This initial position contains an NLOS bias error. However, the NLOS bias is estimated to recalculate the MN position in later steps, which is explained in the next section.

If the bias vector **b**(*k*) is known, the optimal estimate **X̂**(*k*) is given by [16]:

(11) $$\hat{\text{X}}(k)={\left({\text{H}}_{0}^{T}{\text{R}}^{-1}{\text{H}}_{0}\right)}^{-1}{\text{H}}_{0}^{T}{\text{R}}^{-1}\text{y}(k)-{\left({\text{H}}_{0}^{T}{\text{R}}^{-1}{\text{H}}_{0}\right)}^{-1}{\text{H}}_{0}^{T}{\text{R}}^{-1}\text{b}(k)=\tilde{\text{X}}(k)+\text{Ub}(k)$$

where $\text{U}=-{\left({\text{H}}_{0}^{T}{\text{R}}^{-1}{\text{H}}_{0}\right)}^{-1}{\text{H}}_{0}^{T}{\text{R}}^{-1}$ is a bias correction matrix, and $\tilde{\text{X}}(k)={\left({\text{H}}_{0}^{T}{\text{R}}^{-1}{\text{H}}_{0}\right)}^{-1}{\text{H}}_{0}^{T}{\text{R}}^{-1}\text{y}(k)$ stands for the bias-free position estimate.

However, in reality, **b**(*k*) is unknown and has to be estimated. In order to estimate **b**(*k*) from (9), the observed bias metric is defined as:

(12) $$\begin{array}{lll} \text{r}(k) & = & \text{y}(k)-{\text{H}}_{0}\tilde{\text{X}}(k)=({\text{H}}_{0}\text{X}(k)+\text{b}(k)+\text{n}(k))-{\text{H}}_{0}(\hat{\text{X}}(k)-\text{Ub}(k))\\ & = & \left(\text{I}+{\text{H}}_{0}\text{U})\text{b}(k)+{\text{H}}_{0}(\text{X}(k)-\hat{\text{X}}(k))+\text{n}(k\right)\\ & = & \text{L}\cdot \text{b}(k)+\text{v}(k) \end{array}$$

where **L** = **I** + **H**_0_**U**, and the bias noise is given by:

(13) $$\text{v}(k)={\text{H}}_{0}\left[\text{X}(k)-\hat{\text{X}}(k)\right]+\text{n}(k)$$

Then, the following constrained optimization problem is defined to estimate the NLOS bias errors:

(14) $$\begin{array}{l} \hat{\text{b}}(k)=\text{arg}\;\text{min}{\left(\text{r}(k)-\text{L}\cdot \text{b}(k)\right)}^{T}{\text{Σ}}_{\text{v}}^{-1}(\text{r}(k)-\text{L}\cdot \text{b}(k))\\ s.t.\;b_{i}(k)\in \left(b_{i}^{L}(k),b_{i}^{U}(k)\right),\;i=1,2\cdots M \end{array}$$

where **Σ_v_** = *E*[**v**(*k*)**v**(*k*)*^T^*] is a covariance matrix of the error **v**(*k*), and $\left(b_{i}^{L}(k),b_{i}^{U}(k)\right)$ are sets in which each bias error *b_i_*(*k*) between a MN and the *i*th FN lies. For the TOA measurement, the lower bound $b_{i}^{L}(k)$ always satisfies $b_{i}^{L}(k)\geq 0$, and the upper bound $b_{i}^{U}(k)$ may be produced based on the geometrical layout of the MN and the *i*th FN. Specifically, we may set the upper bound by [17]:

(15) $$b_{i}^{U}(k)=\text{min}\left\{z_{i}(k)+z_{j}(k)-l_{\text{ij}},\;i,j=1,\cdots ,M,\;j\neq i\right\}$$

where *l_ij_* is the distance between the *i*th FN and the *j*th FN.

It is obvious that (14) is a constrained LS problem, a type of quadratic programming (QP) problem. There are many algorithms developed to solve this type of problem [18]; here, the Matlab function quadprog is used to find the solution.

## 4. EKF Tracking Algorithm with NLOS Correction

Assume that a MN of interest moves on a two-dimensional plane, and the motion state at time instant *k* is defined as the vector **S**(*k*) = [*x*_*k*_, *y*_*k*_, *ẋ*_*k*_, *ẏ*_*k*_]*^T^*, where [*x*_*k*_, *y*_*k*_] corresponds to the horizontal and vertical Cartesian coordinates of the mobile position, [*ẋ*_*k*_, *ẏ*_*k*_] are the corresponding velocities. The mobile state with random acceleration can be modeled as:

(16) $$\left[\begin{array}{c} x_{k}\\ y_{k}\\ {\dot{x}}_{k}\\ {\dot{y}}_{k} \end{array}\right]=\left[\begin{array}{cccc} 1 & 0 & \Delta t & 0\\ 0 & 1 & 0 & \Delta t\\ 0 & 0 & 1 & 0\\ 0 & 0 & 0 & 1 \end{array}\right]\left[\begin{array}{c} x_{k-1}\\ y_{k-1}\\ {\dot{x}}_{k-1}\\ {\dot{y}}_{k-1} \end{array}\right]+\left[\begin{array}{cc} \frac{\Delta t^{2}}{2} & 0\\ 0 & \frac{\Delta t^{2}}{2}\\ \Delta t & 0\\ 0 & \Delta t \end{array}\right]\left[\begin{array}{c} w_{x_{k}}\\ w_{y_{k}} \end{array}\right]$$

where Δ*t* is the discrete sampling period, and the random process **W**(*k*) = [*w*_*x*_*k*__, *w*_*y*_*k*__]*^T^* is a 2 × 1 vector. Since **W**(*k*) is a white noise, *E*[**W**(*k*)**W***^T^*(*k* + *j*)] = 0 for *j ≠* 0. The covariance matrix **Q** of **W**(*k*) is $\text{Q}=\mathrm{diag}\left(\sigma_{x}^{2},\sigma_{y}^{2}\right)$. The vector form of (16) can be described as:

(17) S(k)=ΦS(k−1)+ΓW(k)

Because of the non-linear measurement equation, the EKF has to be used. Applying the linearization method used in EKF design, the linearized measurement equation becomes:

(18) z(k)=F(k)S(k)+n(k)+b(k)

where:

(19) $$\text{F}(k)={\left[\begin{array}{cccc} \partial f_{1}/\partial x_{k} & \partial f_{1}/\partial y_{k} & \partial f_{1}/\partial {\dot{x}}_{k} & \partial f_{1}/\partial {\dot{y}}_{k}\\ \vdots & \vdots & \vdots & \vdots \\ \partial f_{M}/\partial x_{k} & \partial f_{M}/\partial y_{k} & \partial f_{M}/\partial {\dot{x}}_{k} & \partial f_{M}/\partial {\dot{y}}_{k} \end{array}\right]}_{{}_{\text{S}=\hat{\text{S}}(k)}}$$

Similar to the Kalman filter, the operations of the EKF can be represented by two recursive steps. The prediction step includes the following operations:

(20) S(k|k−1)=ΦS(k−1|k−1)

(21) $$\text{P}(k|k-1)=\text{ΦP}(k-1|k-1){\text{Φ}}^{T}+\text{ΓQ}{\text{Γ}}^{T}$$

(22) $$\text{K}(k)=\text{P}(k|k-1){\text{F}}^{T}(k-1){\left[\text{F}(k-1)\text{P}(k|k-1){\text{F}}^{T}(k-1)+\text{R}(k)\right]}^{-1}$$

where **K**(*k*) is Kalman gain and **P**(*k* | *k* − 1) is the covariance matrix of **S**(*k* − 1| *k* − 1). The covariance matrix **R**(*k*) is updated by the adaptive approach in [19]. The measurement correction step is written as follows:

(23) P(k|k)=[I−K(k)F(k−1)]P(k|k−1)

(24) S(k|k)=S(k|k−1)+K(k)[z(k)−F(k−1)S(k|k−1)]

Although the EKF is probably the most widely used estimation algorithm for nonlinear systems, it is derived basing on Gaussian noise condition. In the LOS environments where only measurement noise is considered, the EKF is an optimal estimator and can improve the tracking accuracy since measurement noise is usually assumed to obey Gaussian distribution [7,20]. For the NLOS error which depends on the environmental conditions, the bias term *b_i_*(*k*) was modeled in different ways in the literature, such as exponentially distributed [21,22], uniformly distributed [23,24], Gaussian distributed [25], or based on an empirical model from measurements [26,27]. Therefore, it is difficult to accurately estimate the MN location in the NLOS scenario using the conventional EKF, and at worst the EKF may cause its estimates to diverge. This paper proposes an efficient and practical method to facilitate an accurate EKF tracking. The main idea behind the proposed approach is to get rid of the NLOS bias directly in the updating process.

Once the NLOS bias is obtained in Section 3, the measurement updating Equation (24) is modified as:

(25) $$\text{S}(k|k)=\text{S}(k|k-1)+\text{K}(k)\left[\text{z}(k)-\text{F}(k-1)\text{S}(k|k-1)-\hat{\text{b}}(k)\right]$$

where **b̂**(*k*) is the estimation of NLOS propagation delay by means of the optimization method. Since the NLOS bias is eliminated directly in (25), the modified EKF can mitigate the adverse effects caused by the NLOS error. And thus the modified EKF overcomes the shortcomings of the conventional EKF that is only fit to the Gaussian noise.

## 5. NLOS Identification

The unique change of the modified EKF is to subtract the NLOS bias in the updating equation, so increased computing time is small, but the NLOS bias estimation using the optimization method will increase the computational complexity. In the mixed LOS/NLOS environments, however, the above NLOS bias estimation is actually needless when the LOS situation appears. However, we do not know when and how often the LOS or NLOS conditions appear and disappear in the realistic scenarios, so it is important to check the LOS/NLOS condition for reducing unnecessary computing time. In the previous algorithms [10,11], identification is implemented by a time-history based hypothesis test, which need a period of samples to calculate the standard deviation of the measured range data. In this work, the main feature behind the proposed approach is real-time identification, since only the present innovation vector and its covariance matrix are used.

From the incoming measurement and the optimal prediction obtained in the previous step, the innovation sequence in the LOS case is defined as:

(26) $$\text{α}(k)=\text{z}(k)-\hat{\text{z}}(k|k-1)=\text{F}(k)\tilde{\text{S}}(k|k-1)+\text{n}(k)$$

where **ẑ**(*k* | *k* − 1) = **F**(*k*)**S**(*k* | *k* − 1) is the estimate of the one-step predicted measurement values, and **S̃**(*k* | *k* − 1) = **S**(*k*) − **S**(*k* | *k* − 1) is the one-step prediction error. Due to the white Gaussian noise presumption in the LOS condition, the theoretical covariance matrix of **α**(*k*) is:

(27) $${\text{D}}_{\text{LOS}}(k)=E\left\{\text{α}(k){\text{α}}^{T}(k)\right\}=\text{F}(k)\text{P}(k|k-1){\text{F}}^{T}(k)+\text{R}(k)$$

But in the NLOS case, the measurement equation becomes:

(28) z(k)=F(k)S(k)+n(k)+b(k)

Correspondingly, the innovation vector turns into:

(29) $$\text{α}(k)=\text{F}(k)\tilde{\text{S}}(k|k-1)+\text{n}(k)+\text{b}(k)$$

Assuming that the covariance matrix of the NLOS error **b**(*k*) is defined as **o**(*k*), the covariance matrix of **α**(*k*) in the NLOS condition can be expressed as:

(30) $${\text{D}}_{\text{NLOS}}(k)=E\left\{\text{α}(k){\text{α}}^{T}(k)\right\}={\text{D}}_{\text{LOS}}(k)+\text{o}(k)$$

From (29,30), the positive deviation will be added in the innovation vector and its covariance matrix in the NLOS conditions, since the NLOS propagation generally leads to a positive bias in the estimation range. Some canonical field tests in [5,28–29] have shown that the positive bias in the TOA measurement caused by the NLOS error can typically be of up to several hundred meters in the outdoor environment. For example, the means of the NLOS errors in GSM system tested by the Nokia corporation and in IS-95 CDMA system tested by the Korea Telecom corporation are on the order of 513 m and 589 m, respectively [5,28]. On the other hand, the indoor environments are full of obstacles, walls, and other objects, which affect transmitted signal, and then range estimates might be much larger than the true distances. Therefore, the large deviation value can be used to distinguish the LOS/NLOS situations.

Because variance calculation needs a time series of range measurements from each FN, the LOS/NLOS identification based on variance comparison is not suitable for real-time application. On the contrary, the square sum of the innovation vector, *i.e.*, **α***^T^*(*k*)**α**(*k*), which still contains the estimation error information of the range measurement, can be used to indicate the NLOS error is present or not. When the MN has a LOS path to the FN, then the square sum of the innovation vector in theory can be obtained by:

(31) $${\text{α}}^{T}(k)\text{α}(k)=Tr\left[\text{F}(k)\text{P}(k|k-1){\text{F}}^{T}(k)+\text{R}(k)\right]$$

in which *Tr*[] denotes the trace of a matrix.

If not, the NLOS error is present, then we would expect for the square sum of the innovation vector to have a significantly larger deviation than the values of (31). This assumption is strongly supported by the field test results and the above analysis, which clearly indicate that the presence of the NLOS error increases the deviation of the measurements in a significant manner.

**Figure 1.**
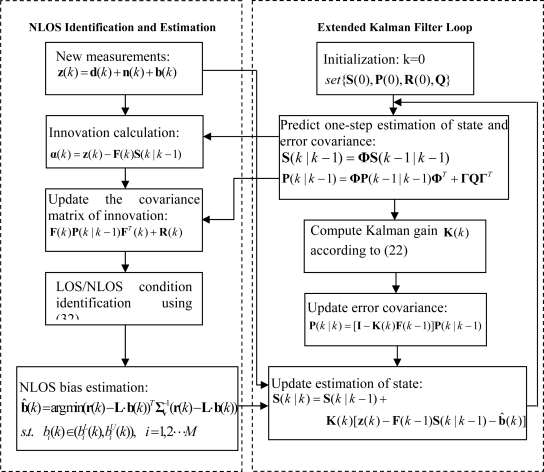
Flow chart of the proposed algorithm with NLOS correction.

Based on the calculated square sum of the innovation vector, therefore, the hypothesis testing can decide whether NLOS components exist. The decision rule of the LOS/NLOS hypothesis testing for mobile location is chosen as follows:

(32) $$\begin{array}{l} H_{0}:{\text{α}}^{T}(k)\text{α}(k)\leq \gamma Tr[\text{F}(k)\text{P}(k|k-1){\text{F}}^{T}(k)+\text{R}(k)],\;\text{LOS}\;\text{condition}\\ H_{1}:{\text{α}}^{T}(k)\text{α}(k)>\gamma Tr[\text{F}(k)\text{P}(k|k-1){\text{F}}^{T}(k)+\text{R}(k)],\;\text{NLOS}\;\text{condition} \end{array}$$

where the scaling factor *γ* ≥ 1 is used to reduce the probability of false detection, and the value of *γ* = 1.1 is chosen in this paper. Unlike the previous algorithms in [10,11], the proposed method can satisfy most real-time applications, because only current **α**(*k*), **F**(*k*), **P**(*k*|*k*−1) and **R**(*k*) are needed and they can be updated in time according to the EKF. To sum up, the proposed algorithm is summarized in Figure 1.

## 6. Simulation Results

Simulation results are provided in this section to assess the performance of the proposed algorithm in both large-scale and small-scale environments. Two conditions in [10,13] are considered to simulate the large-scale mobile communication environments, whereas some results in small-scale conditions will be presented in Section 6.4. The first case in the large-scale environment is with the fixed LOS/NLOS condition, and the second case is with the LOS/NLOS transition condition which the LOS or NLOS of each FN is changed for each 200 samples in an alternate way. Many NLOS mitigation algorithms can meet the FCC location requirement when seven FNs are available. This is because while many FNs (e.g., seven) increase the chance of mutual NLOS error canceling, few NLOS FNs (e.g., three) increase the tendency to greatly bias the final location estimate [30]. In a more realistic scenario there are often only three FNs detectable from a MN, so we assume that the MN can receive the signals from three FNs in both cases. The coordinates (in meters) of these three FNs are (0, 0), (8,600, 0) and (4,300, 7,500). The simulated measurement data are generated by adding the measurement noise and the NLOS noise to the true distance from a MN to each FN. In comparison with the NLOS error, the measuring error is usually considered to be several tens of meters at the most when super-resolution TOA estimation and high precision synchronization techniques are used. In this paper, the measurement noise is assumed to be a white Gaussian random variable with zero mean and standard deviation 50 m, which comprises the synchronization errors, clock drifts errors, and so on [20,31]. For simplicity, we simulate the MN moves in a straight line at a constant speed of (10 m/s, 15 m/s). The random acceleration variance $\sigma_{x}^{2}$, $\sigma_{y}^{2}$ are both chosen to 1 *m/s*^2^. The simulated trajectory has L = 2,000 time samples, and the sample interval *Δt* = 0.1 *s.* We compare the performance of the EKF, the Iterated NLOS EKF [15] and the proposed method in the same conditions. All the simulation results are obtained based on N = 100 Monte Carlo realizations with the same parameters. The mobile location error is calculated with the elimination of the first 100 samples so as to ignore the large location error caused by the initial conditions.

### 6.1. In the Fixed LOS/NLOS Condition

In this condition, the NLOS measurement noise is fixed for the whole trajectory, which is assumed to be a white Gaussian random variable with positive mean *m_NLOS_* = 513 m, and standard deviation *σ_NLOS_* = 436 m [5,13].

Table 1 shows the root mean square error (RMSE) of the three algorithms in four different cases under this condition. Assuming the true MN location is (*x*_*s*_, *y*_*s*_), the RMSE at the time instant k is defined as ${\mathrm{RMSE}}_{k}=\sqrt{\frac{1}{N}\sum_{i=1}^{N}\left[{\left(x_{k,i}-x_{s}\right)}^{2}+{\left(y_{k,i}-y_{s}\right)}^{2}\right]}$.

It can be seen from Table 1 that, the proposed method achieves the least RMSE among all the three methods, and the EKF method and the Iterated NLOS EKF algorithm can not meet the FCC requirement (for 67% location error at 100 m and 95% location error at 300 m) even if there is only one NLOS FN. Even in the worst case, *i.e.*, when the three FNs are all in NLOS condition, the proposed method has the performance with 67% error at 37.37 m and 95% location error at 76.58 m, which is far below the location requirement mandated by the FCC.

**Figure 2.**
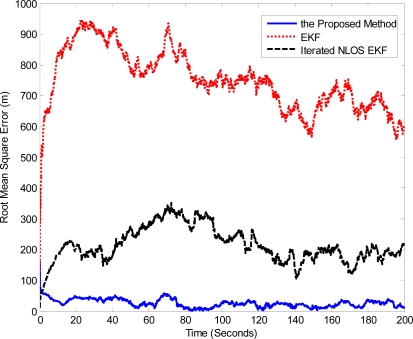
Comparison of the RMSE when the three FNs are all in NLOS conditions.

In order to evaluate the tracking precision obtained by the proposed algorithm, we have computed the RMSE of the three different trackers in the worst situation, *i.e.*, no LOS path between the MN and each FN. We can observe in Figure 2 that the EKF method results in poor tracking performance in the NLOS conditions. Since the iterated NLOS EKF algorithm utilizes the NLOS bias estimation and correction, its location estimation error is much smaller than that of the EKF method, but it is clearly outperformed by the proposed algorithm in terms of RMSE. These results reveal that our method is very robust to bad propagation environments and effectively enhances location accuracy.

### 6.2. In the LOS/NLOS Transition Condition

**Figure 3.**
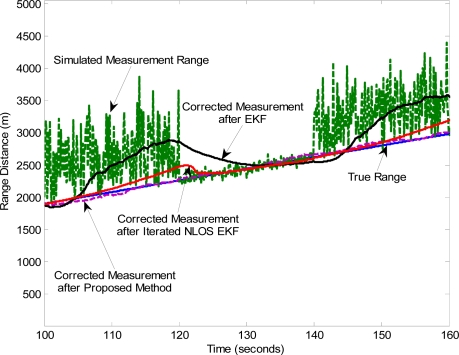
Zoom of the range estimation by three algorithms during 60 seconds.

This group of simulations will show the great improvement in the accuracies on the range estimations and tracking trajectories in the LOS/NLOS transition condition. In order to do that, we take TOA measurements during 60 seconds in three intervals NLOS-LOS-NLOS. Figure 3 shows how the proposed algorithm works for TOA measurements in the mixed LOS/NLOS environment. Simulation results show the proposed method can closely track the true distance between the MN and the corresponding FN, while the estimation of the EKF is far away from the true distance with a big positive bias in the NLOS condition. Although the iterated NLOS EKF algorithm performs as good as the proposed method in the LOS phase, its performance is still much worse than that of the proposed method in the NLOS situation.

**Figure 4.**
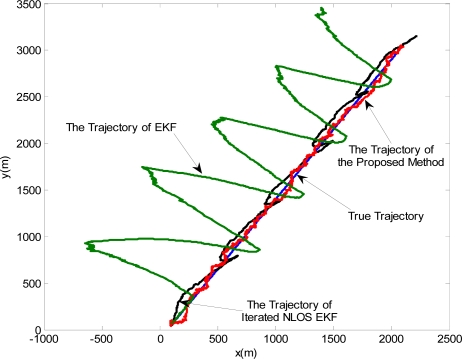
The estimated trajectories of the MN by three algorithms from a single realization.

Accordingly, it can be seen from the estimated trajectories in Figure 4 that the proposed algorithm can provide better tracking capability comparing with the other two schemes. Although the Iterated NLOS EKF method improves the accuracy of the location estimation comparing with the conventional EKF, it still severely deviates from the true trajectory.

**Figure 5.**
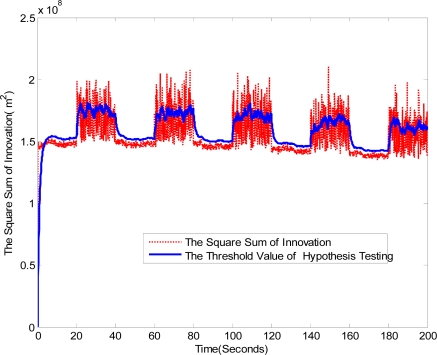
The identification results of the LOS/NLOS hypothesis testing.

The identification results in the LOS/NLOS mixed environment is provided in Figure 5. Because the innovation vector contains the estimation error information of the range measurement which represents a kind of additional information available to the filter as a consequence of the new observation, the proposed identification method according to (32) can immediately and effectively respond to the LOS/NLOS changes. Moreover, it can be seen form Figure 5 that the changes of the LOS/NLOS situation can be identified accurately, except several false points in the beginning moment. The accurate identification of LOS/NLOS conditions will enhance location accuracy and reduce unnecessary computing time, when the proposed algorithm is implemented.

### 6.3. Complexity Analysis and Comparison

Compared with the conventional EKF smoother, the additional computation in the proposed algorithm is primarily introduced by the estimation of NLOS bias using constrained LS optimization, which mainly involves the operations of matrix inverse and multiplication. The complexity of the matrix inverse operation is *O*(*N*^3^), and the multiplication operation is *O*(*N*^2^), where *N* represents the dimension of the matrix. Table 2 shows the complexity comparison results of the three algorithms in terms of the actual computation time. The total sampling times *L* in all three methods equal to 2,000. The experiments are processed on the computer with Pentium IV 2.4 GHz processor and 1 GB memory.

From Table 2, it shows that the computer running time of the proposed method is about 0.16 second larger than the conventional EKF and 0.14 second larger than the Iterated NLOS EKF. Considering the large performance gain that the proposed method achieves, this slight complexity is totally acceptable. Note that the computer time is the results of 2,000 iterations, so the average compute time in a single iteration is small. Therefore, the proposed method is promising for most real-time applications.

### 6.4. Accuracy Evaluation in a Short-Range Environment

To verify the proposed method and examine the performance in a short-range environment, the following simulations are carried out. The location area is a square with dimensions of 100 *×* 100 m. In the first case, four FNs are assumed to be located at (0, 0), (100, 0), (0, 100), and (100, 100) (in meters), respectively. This is based on the well-known fact that placing the FNs along the boundary of the location area produces better performance. On the contrary, the four anchors’ locations are almost-collinear in the second case, at (0, 0) (38, 39), (80, 81), and (100, 100) (in meters), which is an extremely bad node geometry. The measurement noise is Gaussian with zero mean and a standard deviation σ*_i_* being proportional to the distance, *i.e.*, σ*_i_* = 0.02*d_i_*. The NLOS error is exponentially distributed with the parameter denoted by *λ_i_* = 0.08*d_i_*, and hence the mean and variance of NLOS error are *λ_i_* and $\lambda_{i}^{2}$, respectively. The selection of these values is in accordance with the range estimation accuracy when using TOA measurements in [32,33]. We simulate the MN moves in a straight line at a constant speed of (0.1 m/s, 0.15 m/s). The random acceleration variance $\sigma_{x}^{2}$, $\sigma_{y}^{2}$ are both chosen to 0.01 m/s^2^. The simulated trajectory has L = 200 time samples, and the sample interval Δ*t* = 0.1*s*.

**Figure 6.**
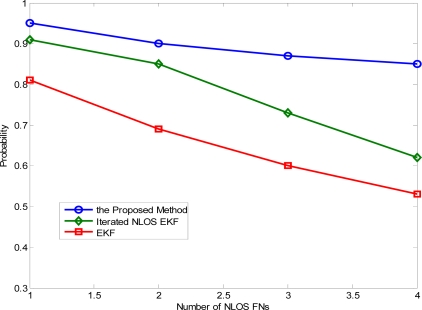
Impact of the number of NLOS FNs on the location accuracy in the good node geometry.

Figure 6 illustrates the effect of the number of NLOS FNs (from one to four) when the location error is up to 5 m under the good node geometry. Basically, the accuracy degrades, approximately in a linear form, as the number of NLOS FNs increases. The proposed method achieves accuracy of 5 m at a probability greater than 85% in all the cases, while the iterated NLOS EKF algorithm performs only well when there is no more than two NLOS FNs.

**Figure 7.**
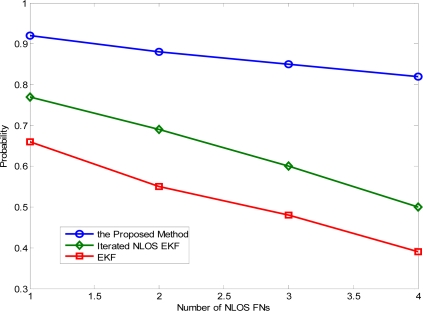
Impact of the number of NLOS FNs on the location accuracy in the bad node geometry.

On the other hand, Figure 7 shows the probability of the location error versus the number of NLOS FNs in the bad node geometry when the location error is up to 5 m. As expected, the probability goes down as the number of NLOS FNs increases. Apparently, the iterated NLOS EKF algorithm and the conventional EKF approach perform very poorly in the bad anchor geometry. However, the accuracy of the proposed method drops only about 3% under the given anchor geometry in the second case. This may indicate that, in practice, whenever the node geometry can be obtained, the proposed algorithm may be applied to produce an accurate location estimate.

## 7. Conclusions

Because the NLOS propagation is ubiquitous in both indoor and outdoor positioning scenarios, a robust algorithm is required to mitigate the impact of the NLOS location estimation error. In this work, we have developed a suitable NLOS identification and mitigation algorithm for the improvement of location accuracy in EKF-based mobile location. Simulation results show that the proposed algorithm exceeds the FCC target and significantly outperforms the other two methods. Meantime, it does not depend on a particular distribution of the NLOS error. As compared to the conventional time-history method, the proposed identification approach has achieved the real-time capability by using only current innovation sequence. The complexity comparison suggests that the performance gain of the proposed method is at the expense of increasing the computer time. However, the difference of complexity is small and acceptable when considering the large performance gain it achieves. This method can still be considered as a candidate for most real-time applications. Further investigation will emphasize on the impact of different motion models and channel models for the proposed algorithm and the theoretic bound on the location estimation precision for a given set of measurements.

## Figures and Tables

**Table 1.** Performance comparisons among three algorithms under the different NLOS conditions.

**Algorithm**	**The proposed method**		**Iterated NLOS EKF**		**EKF**	

Error (m)	67%	95%	67%	95%	67%	95%
3LOS, 0NLOS	17.17	30.07	34.58	73.35	9.83	38.39
2LOS, 1NLOS	32.76	63.96	105.8	221.1	309.2	375.1
1LOS, 2NLOS	35.99	69.52	238.5	280.1	789.9	859.5
0LOS, 3NLOS	37.37	76.58	301.9	315.9	828.5	916.3

**Table 2.** Computer running time of the three methods.

**Method**	**The proposed method**	**Iterated NLOS EKF**	**EKF**
Average Time(s)	0.352	0.216	0.191
Standard deviation(s)	0.011	0.012	0.016
